# Importation of Human Seoul Virus Infection to Germany from Indonesia

**DOI:** 10.3201/eid2406.172044

**Published:** 2018-06

**Authors:** Jörg Hofmann, Sabrina Weiss, Martin Kuhns, Annekathrin Zinke, Heike Heinsberger, Detlev H. Kruger

**Affiliations:** Charité–University of Medicine Institute of Virology, Berlin, Germany (J. Hofmann, S. Weiss, D.H. Kruger);; Humboldt University, Berlin (J. Hofmann, S. Weiss, D.H. Kruger);; Medilys Laborgesellschaft mbH, Hamburg, Germany (M. Kuhns);; Asklepios Hospital Harburg, Hamburg (A. Zinke, H. Heinsberger)

**Keywords:** Seoul virus, SEOV, viruses, hantaviruses, importation, imported case, human infection, hemorrhagic fever with renal syndrome, HFRS, zoonoses, Germany, Indonesia

## Abstract

Seoul hantavirus–associated hemorrhagic fever with renal syndrome cases are rare outside Asia and have not yet been found in Germany. We report clinical and molecular evidence for a Seoul virus infection in a patient in Germany. The infection was most likely acquired during a stay in Sulawesi, Indonesia.

Hantaviruses are globally emerging zoonotic pathogens that cause hemorrhagic fever with renal syndrome (HFRS) and hantavirus cardiopulmonary syndrome (*1*). One representative of this negative-sense, single-stranded RNA virus family is rat-associated Seoul virus (SEOV). In comparison to infections by prototypical Hantaan virus (HTNV), infection with SEOV is believed to lead to somewhat milder disease with shorter clinical phases (*2*). The most characteristic manifestations of SEOV infection are prominent abdominal symptoms, including hepatomegaly, hepatic dysfunction, and mild renal failure (*2*). A clear distinction from infections by related hantaviruses, such as HTNV, by serodiagnostic means is difficult and complicates interpretation of current clinical studies on (putatively) SEOV-infected patients.

SEOV infections have been found in rats and humans mainly in Asia but also worldwide (*3*). In Europe, molecular analysis has shown circulation of SEOV in wild brown rats (*Rattus norvegicus*) and in pet rats in the United Kingdom, France, Belgium, the Netherlands, and Sweden; however, human SEOV infections have been diagnosed by using only serologic analysis (*4*). Unequivocal molecular proof of human SEOV infections in Europe has been shown only for 4 patients in France (*5*) and 1 patient in the United Kingdom (https://www.gov.uk/government/publications/hantavirus-infection-in-people-sero-surveillance-study-in-england).

In Germany, SEOV-specific antibodies or SEOV RNA have not been detected in rats or humans. We report on a case of molecularly proven hantavirus disease caused by SEOV infection in a patient in Germany who probably acquired the infection in Indonesia.

## The Patient

On April 25, 2017, a 70-year-old man from Germany visited the emergency department at Asklepios Klinik Harburg (Hamburg, Germany) and reported severe diarrhea, thoracic/back pain, and bronchopulmonary symptoms. He also reported a fever that emerged at the end of a multiweek vacation on the island of Sulawesi in Indonesia a few days before his return to Germany on April 12. On May 2, he was hospitalized because of acute kidney injury.

We obtained laboratory findings for blood samples collected during the inpatient period of 11 days (Table). An initially low platelet count (66/nL) at admission returned to a reference value at day 8 of hospitalization. Serum creatinine levels were increased; maximum values were observed at days 2 and 3 of hospitalization. The glomerular filtration rate was decreased. Leukocyte counts and levels of C-reactive protein and lactate dehydrogenase were slightly increased. Increased levels of liver enzymes (aspartate aminotransferase, alanine aminotransferase, and γ-glutamyltransferase) indicated hepatic involvement, a characteristic of SEOV infections (*2*). Diuresis returned to reference values during hospitalization, and no polyuria was observed. The patient was discharged from the hospital with a serum creatinine level of 1.8 mg/dL and in largely normalized general condition.

**Table:** Biochemical parameters of a case-patient during 11 days of hospitalization who was infected with Seoul virus imported to Germany from Indonesia*

Parameter	Reference range	Day 1	Day 2	Day 3	Day 9	Day 11
Platelet count	160–370/nL	66	85	112	394	490
Creatinine	0.7–1.2 mg/dL	3.7	5.5	5	2.3	1.8
GFR CKD-EPI	>60 mL/min	16	10	11	28	37
Leukocyte count	3.5–9.8 cells/nL	12.8	15.7	16.8	12.1	11.7
CRP	<5.0 mg/L	54.4	ND	26.5	10.4	ND
LDH	<250 U/L	735	561	ND	285	ND
GGT	<60 U/L	137	115	121	159	ND
ALT	<50 U/L	75	119	234	120	ND
AST	<50 U/L	ND	173	ND	46	ND
Urea	18–55 mg/dL	ND	240	ND	73	48
Hemoglobin	13.5–17.5 g/dL	17.6	16	15.1	12.9	11.4

*ALT, alanine aminotransferase; AST, aspartate aminotransferase; CKD-EPI, chronic kidney disease epidemiology collaboration; CRP, C-reactive protein; GFR, glomerular filtration rate using the CKD-EPI formula; GGT, γ-glutamyltransferase; LDH, lactate dehydrogenase; ND, not determined.

We performed initial laboratory diagnostics (serologic analysis for hantavirus) by using the Hantavirus Profile 1 Immunoblot (Euroimmun, Lübeck, Germany) in the hospital laboratory and the *recom*Line HantaPlus IgG and IgM assays (Mikrogen, Martinsried, Germany) in our laboratory. The Profile 1 blot does not contain SEOV antigen, but serum from the patient showed reactivity for Dobrava-Belgrade virus (DOBV) IgG and HTNV IgM. The *recom*Line IgG and IgM blots showed reactivity for DOBV, HTNV, and SEOV; the weakest result was for SEOV nucleocapsid protein.

For molecular typing, we tested the first blood sample collected during hospitalization by using reverse transcription for hantavirus RNA. We obtained partial sequences of genes coding for hantavirus polymerase (large [L] RNA segment) by using HAN-L primers (*6*). We also obtained nucleocapsid protein (small [S] RNA segment) sequences by using primers S1 and S2 (*7*) and primers S598 (5′-ATG AAG GCA GAA GAG ATT ACA CC[TA] GG-3′) and S6HC (5′-CCA GCA AAC ACC CAT ATT GAT GAT-3′) as nested primers. Sequences of the strain Sulawesi-01/17 were deposited in GenBank under accession nos. MG386252 for the L segment and MG386253 for the S segment. We showed by phylogenetic analysis that L and S sequences obtained from the patient segregated to the main cluster of SEOV strains, which clearly demonstrated that SEOV was the causal agent of infection (Figure).

**Figure:**
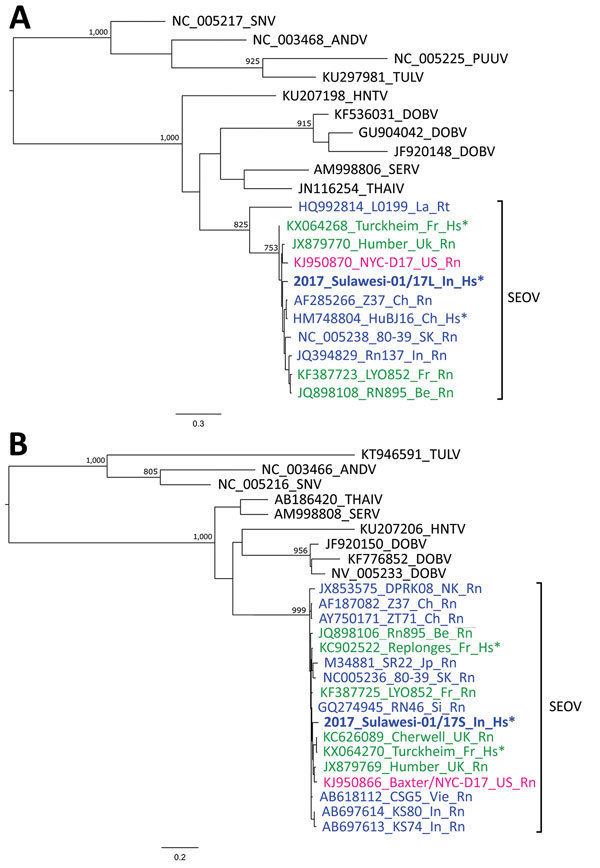
Maximum-likelihood phylogenetic trees of partial RNA segments of orthohantaviruses. A) Large RNA segments based on a 347-nt alignment and the general time reversible plus gamma distribution model of nucleotide substitution. B) Small RNA segments based on a 318-nt alignment and the Hasegawa–Kishino–Yano 85 plus gamma distribution model. Trees were constructed by using PhyML3.0 (*8*) and the best-fitting model according to smart model selection in this software and 1,000 bootstrap replicates. Values along branches are bootstrap values >75% for major clades. GenBank accession number, strain, country of origin, and host are shown for each virus isolate. Bold indicates SEOV isolated from the patient in this study. Blue indicates SEOV strains from Asia, green indicates SEOV strains from Europe, and red indicates SEOV strains from the Americas. Scale bars indicate nucleotide substitutions per site. *Sequences from viruses of human origin. ANDV, Andes virus; Be, Belgium; Ch, China; DOBV, Dobrava-Belgrade virus; Fr, France; HNTV, Hantaan virus; Hs, *Homo sapiens*; In, Indonesia; Jp, Japan; La, Laos; NK, North Korea; PUUV, Puumala virus; Rn, *Rattus norvegicus*; Rt, *R. tanezumi*; SEOV, Seoul virus; SERV, Serang virus; Si, Singapore; SK, South Korea; SNV, Sin nombre virus; THAIV, Thailand virus; TULV, Tula virus; UK, United Kingdom; US, United States; Vie, Vietnam.

## Conclusions

We report an infection with SEOV in a patient in Germany. If one considers the clinical course, this case of HFRS appeared moderate, and the outcome for this case-patient was favorable and showed a full recovery. As expected for SEOV-associated hantavirus disease (*2*), severe gastrointestinal symptoms and liver involvement were observed, but kidney dysfunction was mild and no hemodialysis was needed. However, the clinical course appeared unusually protracted (≈6 weeks) between the febrile phase and discharge from hospital. Typing of the causative hantavirus by using 2 commercial immunoblots was misleading, but sequence data obtained from the L and S segments unequivocally confirmed that the patient was infected with SEOV.

Molecular phylogenetic analysis of genetically characterized SEOV strains resulted in creation of 4 phylogroups (*9*). Most strains, including all strains from locations other than mountainous areas of China, belong to major phylogroup A and have probably spread from Asia as a result of distribution of rats during trade activities by humans (*9*). Although SEOV strains are found in ports and countries with overseas traffic, no SEOV infections of rats or humans have been reported in Sulawesi in Indonesia. SEOV RNA in rats, but no proof of human infections, was reported in Jakarta, on the island of Java in Indonesia (*10**,**11*).

Because of the wide spread of SEOV caused by extensive movement of its natural host (rats), analysis of the nucleotide sequence of the SEOV strain from our patient does not enable identification of the place of infection. This strain is related to SEOV strains from Asia, Europe, and the United States (Figure). This finding contrasts with those for other hantaviruses, for which there are clear spatial association between virus strains and carrier hosts, such as vole-associated Puumala virus (*6*,*12*).

Although molecular characterization of the SEOV strain isolated from the patient enabled diagnosis of SEOV infection, these results did not identify the original place of infection. The clinical course, including onset of disease, makes it highly probable that the patient acquired the infection during his stay in Indonesia. Because he did not report any further travel activities during this stay, we conclude that he had acquired the infection in Sulawesi in Indonesia.

In summary, we report a human SEOV infection imported to Germany from Indonesia. Our results demonstrate that extended molecular diagnostics are required for reliable hantavirus typing, especially for patients with travel histories.
